# Severe Leptospirosis Features in the Spleen Indicate Cellular Immunosuppression Similar to That Found in Septic Shock

**DOI:** 10.3389/fimmu.2019.00920

**Published:** 2019-04-30

**Authors:** Amaro Nunes Duarte-Neto, Julio Croda, Carla Pagliari, Francisco Garcia Soriano, Antonio Carlos Nicodemo, Maria Irma Seixas Duarte

**Affiliations:** ^1^Departamento de Patologia, Faculdade de Medicina, Universidade de São Paulo, São Paulo, Brazil; ^2^Disciplina de Emergências Clínicas, Faculdade de Medicina, Universidade de São Paulo, São Paulo, Brazil; ^3^Faculdade de Medicina, Universidade Federal do Mato Grosso do Sul e Fundação Oswaldo Cruz, Campo Grande, Brazil; ^4^Unidade de Terapia Intensiva – Hospital Universitário, Universidade de São Paulo, São Paulo, Brazil; ^5^Departamento de Moléstias Infecciosas e Parasitárias, Faculdade de Medicina, Universidade de São Paulo, São Paulo, Brazil

**Keywords:** leptospirosis, Weil's disease, severe pulmonary hemorrhage syndrome, intensive care, spleen, sepsis-related immunosuppression

## Abstract

**Objectives:** To compare microscopic and immunologic features in the spleens of patients who died of pulmonary hemorrhage and shock caused by leptospirosis (11 cases) or Gram-positive/-negative bacterial septic shock (10 cases) to those from control spleens (12 cases from splenectomy).

**Methodology:** Histological features in the red pulp and white pulp were analyzed using archived samples by a semi quantitative score. Immunohistochemistry was used for the recognition of immune cell markers, cytokines, caspase-3 and *Leptospira* antigens.

**Results:** The control group differed significantly from the leptospirosis and septic shock patients which demonstrate strong similarities: diffuse congestion in the red pulp with a moderate to intense infiltration of plasma cells and polymorphonuclear cells; follicles with marked atrophy; high density of CD20^+^ cells; low density of NK, TCD4^+^ and active caspase-3 positive cells and strong expression of IL-10; leptospirosis patients had higher S100 and TNF-α positive cells in the spleen than the other groups.

**Conclusion:** The results suggest that an immunosuppressive state develops at the terminal stage of severe leptospirosis with pulmonary hemorrhage and shock similar to that of patients with septic shock, with diffuse endothelial activation in the spleen, splenitis, and signs of disturbance in the innate and adaptive immunity in the spleen. The presence of leptospiral antigens in 73% of the spleens of the leptospirosis patients suggests the etiological agent contributes directly to the pathogenesis of the lesions. Our results support therapeutic approaches involving antibiotic and immunomodulatory treatments for leptospirosis patients and suggest that leptospirosis patients, which are usually young men with no co-morbidities, form a good group for studying sepsis and septic shock.

## Introduction

Leptospirosis is the most widespread zoonosis in the world and is caused by pathogenic species of the *Leptospira* genus (1). In Brazil, leptospirosis is a re-emergent infectious disease with a growing incidence rate. In the city of São Paulo, the biggest Brazilian metropolis, leptospirosis had an incidence rate of 1.34-−2.73/100,000 inhabitants and a case-fatality of 8.86–15.61% in the period of 2009–2017 (2).

The clinical presentations of leptospirosis range from a non-specific febrile illness to severe forms. Severe leptospirosis has an estimated incidence of 5–15% and patients can present jaundice, renal failure, myocarditis, meningitis, severe pulmonary hemorrhage syndrome (SPHS), shock, and multiple organ failure (3–5). Little is known about the histological features of the spleens of patients with severe leptospirosis.

Describing briefly, the spleen is a secondary lymphoid organ organized in two morpho-functional compartments: the red pulp and the white pulp. The red pulp is constituted by vascular channels—the sinusoids—lined by endothelial cells and resident macrophages which screen and remove pathogens and soluble antigens from the systemic circulation. The white pulp represents the lymphoid tissue, where the specific immune response to pathogens/antigens occurs. It is constituted by the central arteriole, lymphocytes, and antigen presenting cells (mainly dendritic cells and macrophages). The T lymphocytes are in close contact with the central arteriole, forming the periarteriolar lymphoid sheath, and the B lymphocytes are more external, forming follicles (6, 7).

Cumulative evidence from experimental and human studies show that septic shock develops into an immunosuppressive state (or *sepsis-related immunosuppression*) as it evolves until the death of the host, with disturbances in the different components of innate and adaptive immunities (8–12). Circulating leukocyte and cytokine levels indicate that there also are disturbances in the immune response in severe leptospirosis, such as neutrophils at low levels and with impaired antibacterial function (13–15).

Since features of the white pulp of the spleen can indicate immunosuppression in cases of generalized infection with septic shock (8–10), and given that *Leptospira* are likely to behave similarly to Gram positive/negative bacteria, the aim of this study was to determine if patients with severe leptospirosis (Weil's disease with SPHS and shock) have a histological pattern similar to those who die from septic shock caused by Gram-positive/negative infections. To correlate these data with *Leptospira* infection, we analyzed the presence of *Leptospira* antigens in the spleens of the leptospirosis cases.

Clinical trials for treatment of sepsis often exclude patients with leptospirosis because this infection has so far not been characterized as septic. Our results should support the inclusion of leptospirosis groups in clinical trials for drugs targeted to treat sepsis, such as antibiotics.

Table 1 was compiled based on three current references, describing the main components of the immune system associated with sepsis that were studied here and the molecular markers that identify them (16–18). The discussion about other mediators involved in the pathophysiology of sepsis, such as neutrophils, Treg, chemokines, IL-33, and IL-15 are beyond the scope of this work.

**Table 1:** Main components of the immune system associated with sepsis and the molecular markers that identify them.

**Immune system component**	**General role in the immune system**	**Implicated role in sepsis-related immunosuppression**
Monocytes and macrophages	Monocytes can differentiate into macrophages or dendritic cells in tissues to trigger the immune response. Macrophage functions include: phagocytosis, antigen presentation to T cells and dendritic cells, and cytokine release Innate immunity Macrophages express CD68 (present in all macrophages phenotypes and other cells), CD14, 3A5, F4/80, CD206 (mediates phagocytosis of pathogens)	Endotoxin tolerance (↓expression of TLR), ↓release of pro-inflammatory cytokines, ↓HLA-DR expression causing ↓ antigen presentation), production of anti-inflammatory cytokines (IL-10, IL-4), ↑ susceptibility of the host to secondary infections and worse outcome
NK T cell	Important against viral and intracellular pathogens, cytotoxic antibody-dependent activity. Essential for the initial production of IFN in the immune response Innate immunity. Express CD57, CD94, CD159c	Loss by apoptosis, altered cytotoxic function and cytokine production in response to TLR (↓IFNγ production). Low numbers of NK cells are associated with ↑ mortality
Dendritic cell	Professional antigen presentation cell, bridging innate to adaptive immunity; drives the Th1 response through production of IL-12 and TNFα Expresses S100 (dendritic cell types and other cell types), CD1a, CD11c/MHCII (interdigitating dendritic cells), CD21 (specific for follicular dendritic cells)	Loss by apoptosis; inability to induce Th1 response (T cell anergy), due to ↓HLA-DR expression, ↑IL-10 secretion, ↑Treg proliferation, ↓antigen presentation to T and B cells); low number is associated with ↑ mortality
CD4^+^ T helper cell	Can mature into Th1, Th2, or Th17 cell subsets depending on the type of cytokine stimulation from APC, CD20 interaction, etiologic pathogen and other factors. Th1 cells produce more pro-inflammatory cytokines, associated with pathogen eradication. Th2 cells produce more anti-inflammatory cytokines, leading to anergy and “immunoparalysis” in sepsis Adaptive immunity Express CD4, CD3, CD5, CD28, CD45, CD154	Massive loss by apoptosis, leading to anergy (Th2 polarization), adhesion molecule expression, ↓CD28 expression, ↓TCR diversity
CD8^+^ T cytotoxic cells	Cytotoxic activity Adaptive immunity Express CD8, CD3, CD45	Loss by apoptosis, impairment of cytotoxic function, ↓cytokine release, ↓TCR diversity
B-lymphocytes	Carry out antigen presentation to T lymphocytes and antibody production to eliminate pathogens Adaptive immunity Express CD20, CD19, CD79	Loss by apoptosis, exhaustion of B cells, compromised antibody production
TNFα^*^	Potent pro-inflammatory cytokine released in the early phase of sepsis by macrophages, NK cells and T cells. Involved in Th1 response. Induces activation of neutrophils, monocytes and other effector immune cells; induces phagocytosis, synthesis of acute phase proteins by the liver, activation of endothelial cells (coagulation and inflammation), apoptosis and catabolism fever Essential to eradicate pathogens. Excessive release in the initial phase of infection can cause a hyper-inflammatory (“cytokine storm”) response with excessive tissue damage	In the sepsis-related immunosuppression, it is ↓
IFNγ^*^	Pro-inflammatory cytokine released in the early phase of infection and sepsis by Th1 cells, CD8^+^ T cells and NK cells. Essential for T cell Th1 differentiation. Activates T cells and NK cells. Promotes phagocytosis, expression of MHC I and II, antigen presentation, pro-inflammatory cytokine secretion, production of IgG, oxidative, and nitrosative stress	↓Release in the scenery of sepsis-related immunosuppression
IL-1^*^	Pro-inflammatory cytokine released in the early phase (innate immunity) of infection and sepsis. Produced by fibroblasts, hepatocytes, myeloid, endothelial and epithelial cells. Induces endothelial and hepatocyte activation, inflammation, and fever	↓Release in the scenery of sepsis-related immunosuppression
IL-2^*^	Pro-inflammatory cytokine released in the early phase (innate immunity) of infection and sepsis. Produced by T cells. Induces proliferation and differentiation of T cells, proliferation and activation of NK and B cells	↓Release in the scenery of sepsis-related immunosuppression
IL-6^*^	Pro-inflammatory cytokine released in the early phase of infection and sepsis by myeloid and stromal cells. Promotes inflammation, synthesis of acute phase proteins by the liver and antibody production by B cells. May induce Th2 response	↓Release in the scenery of sepsis-related immunosuppression
IL-8^*^	Pro-inflammatory cytokine released in the early phase of infection and sepsis. Induces inflammation, TNFα production, recruitment of neutrophils and myeloid cells	↓Release in the scenery of sepsis-related immunosuppression
IL-12^*^	Pro-inflammatory cytokine released in the early phase of sepsis by macrophages and dendritic cells after activation by pathogens. Essential for inducing T cell Th1 differentiation. Induces T cells, dendritic cells and NK cells to release IFNγ. ↑cytotoxic activity	↓Production by dendritic cells and lymphocytes in scenery of sepsis-related immunosuppression
IL-4^*^	Anti-inflammatory cytokine released by T CD4^+^ Th2 cells and mast cells. Induces Th2 subset differentiation, inhibition of IFNγ-mediated macrophage activation and isotype switch to IgE	Implied as a mediator to induce sepsis-related immunosuppression
IL-10^*^	Regulatory and Th2 cytokine released by Treg cells, macrophages, dendritic cells and CD4^+^ Th2 cells, with anti-inflammatory action. Inhibits the T cell response	Implied as the main mediator of sepsis-related immunosuppression; inhibits expression of IL-12, co-stimulators and MHC II, negatively influences monocyte production of pro-inflammatory cytokines (TNFα, IL-1β, and IL-6)
TGFβ^*^	Anti-inflammatory cytokine released by Treg and Th2 cells (T cells and macrophages). Inhibits T and B cell proliferation and function, inhibits macrophage activation and function. Induces isotype switch to IgA in B cells, and T cell TH17 and Treg differentiation. Increases collagen synthesis	Implied as a mediator to induce sepsis-related immunosuppression
Apoptotic cells	Programmed cell death of immune cells (NK cells, CD4^+^ and CD8^+^ T cells, B cells and dendritic cells in lymphoid tissues), through both death receptor- and mitochondrial-mediated pathways in sepsis Express activated caspase 3, caspase 9, positive TUNEL	Main mechanism of death of immune cells in sepsis; phagocytosis of apoptotic cells by monocytes, macrophages and dendritic cells induces an anergic CD4^+^ Th2 response, with ↑IL-10, compromising pathogen eradication

*CD, cluster of differentiation; IL, interleukin; NK, natural killer; TLR, Toll-like receptor; TCR, T Cell Receptor; Th, helper T cells; Treg, regulatory T cell*.

* *The tissue expression of cytokines is detected by immunohistochemistry using specific monoclonal antibodies*.

## Materials and Methods

This study is a retrospective histological evaluation of the spleen samples from three different groups of patients (leptospirosis, sepsis and control) obtained by autopsy (leptospirosis and sepsis) or by surgery (control) from the Department of Pathology of the Medical School of the University of São Paulo. The records of the Death Verification Service of the Department of Pathology were reviewed to identify all deaths due to severe leptospirosis with both SPHS and shock during the period between January 1st 1988 and January 1st 2005. All autopsies were performed after written informed consent was given by next of kin according our usual procedures.

The project was approved by the Ethical Committee and Research board of the Clinical Hospital of the University of São Paulo Medical School (registered by CAPPesq ICHC n° 0537/06).

### Case Definition of Leptospirosis, Septic Shock, and Control

The definition of leptospirosis was made by clinical-epidemiologic criteria (exposure to soil and water contaminated with urine from typical leptospirosis reservoirs or exposure to flooding after rainfall and clinical features compatible with leptospirosis) plus any of the following diagnostic confirmations: serologic diagnosis by Immunoglobulin M (IgM) enzyme-linked immunosorbent assay (ELISA) or microscopic agglutination test (MAT) (one single sample >1:800; seroconversion or a 4-fold increase in two samples, one collected at the acute phase and another collected at convalescence); positive culture; typical pathological features of leptospirosis in the necropsies (interstitial nephritis, acute tubular necrosis, disorganization of the liver cell plates, pulmonary hemorrhage) accompanied or not by positive immunohistochemistry to the *Leptospira* antigen. All leptospirosis cases had to fulfill the criteria of septic shock (described below). We excluded cases of leptospirosis in which patients had acquired other bacterial infections during intensive care. Autopsies were carried with a median of 8 h after death (from 7–12 h).

Septic shock patients were assigned according to the septic shock definition valid at admission (19): presence of bacterial infection (proven or not) with a systemic inflammatory response syndrome (SIRS) associated with hypotension (mean arterial pressure < 70 mmHg) requiring vasoactive drug infusion. The cases included in this study also fulfill SOFA and quick SOFA criteria for sepsis definition (20). We excluded patients with immunosuppressive disease or taking immunosuppressive medication. Autopsies were carried out with a median of 8 h after death (from 7–11).

The control group was composed of healthy portions of the spleen of individuals who had abdominal trauma and had undergone laparotomy with splenectomy. We excluded patients who arrived at the emergency room with haemorrhagic shock, patients with immunosuppressive disease or taking immunosuppressive medication.

We collected the following data from charts of all patients: age, gender, APACHEII score, intensive care stay duration and peripheral lymphocyte count. For leptospirosis patients, we collected data for aspartate aminotransferase (AST), alanine transaminase (ALT), total serum bilirubin, platelet count, leukocyte count, serum potassium, creatinine and urea.

The APACHE II is a score system that predicts prognosis of patients in the general intensive care unit (ICU), by including age, previous health conditions and physiologic variables (i.e., vital signs and laboratory exams) and is calculated during the first 24 h of hospitalization in the ICU (21).

Sections 5 μm thick of paraffin-embedded spleen samples were stained with Haematoxylin-Eosin (HE) for morphological analysis.

### Immunohistochemistry

The assay was carried out on 4 μm thick paraffin-embedded spleen samples according to Hsu et al. (22), following a procedure standardized by the Pathology Department of the University of São Paulo Medical School. Briefly, specimens were deparaffinized and hydrated in ethanol, the antigens were retrieved in TRIS/EDTA buffer pH 9.0, for 20 min, at 95°C. The primary antibodies were diluted in 1% bovine albumin solution and incubated overnight at 4°C. Afterwards, the biotinylated secondary antibody and streptavidin-peroxidase complex (Dako, Agilent, Santa Clara, CA, USA,) were applied and specimens were incubated for 30 min, at 37°C. Antibody information is provided in Table 2. 3,3-diaminobenzidine tetrahydroxychloride (Sigma-Aldrich/Merck, Darmstadt, Germany) was used as chromogen and the slides were counterstained with haematoxylin. All reactions were performed with positive and negative controls. Two antibodies (anti CD4 and CD8) required signal amplification using the CSAII kit (Dako, catalog number K1497), according to the manufacturer's instructions.

**Table 2:** Antibody information.

**Antigen**	**Provenance, Catalog number**	**Dilution used in immunohistochemistry**
*Leptospira*	University of São Paulo, Brazil^a^	1:400
CD4	Dako M834	1:1000
CD8	Dako M7103	1:30
CD20	Dako M0755	1:40
CD45	Dako M742	1:100
CD68	Dako M0876	1:30
S-100	Dako Z311	1:100
IL1-β	R&D Systems^b^ AF2001-NA	1:20
IL4	R&D Systems AF204-NA	1:40
IL6	R&D Systems AF206-NA	1:20
IL10	R&D Systems MAB217	1:10
IL12	R&D Systems MAB219	1:10
IFN-γ	R&D Systems MAB285	1:30
TNF-α	R&D Systems AF210MA	1:40
CD57	Immunotech^c^ 1166	1:100
TGF-β	Santa Cruz^d^ SC-82	1:50
Caspase 3	Cell Signaling^e^ 9661 S	1:100
IL2r	Novocastra/Leica^f^ CD25	1:20

a *The polyclonal rabbit antibody was produced and validated by the Tropical Medicine Institute (University of São Paulo, SP, Brazil) and was used to detect Leptospira antigens in the liver and kidney for post-mortem diagnosis according to previous published protocols (3, 30)*.

b *R&D Systems Minneapolis, MN, USA*.

c *Immunotech Marseille, France*.

d *Santa Cruz Dallas, TX, USA*.

e *Cell Signaling, Danvers, MA, USA*.

f *Novocastra/Leica, Wetzlar, Germany*.

In the case of cytokine staining, the assay was modified as follows: before blocking, slices were treated with 0.1% saponin in PBS, for 10 min, and then rinsed with PBS. In addition, after secondary antibody incubation, another incubation with saponin was carried out, followed by rinsing and a second blocking of endogenous peroxidase.

The histological features of the spleens were analyzed as following: A—by the presence or absence (0 = absence, 1 = presence of the event) of: pericapsular/subcapsular hemorrhage, red pulp hemorrhage, red pulp necrosis, vessel necrosis and thrombosis, central artery of the white pulp with signs of endothelial activation, extra medullary hematopoiesis, depletion of T-zone and B-zone, atrophy or hyperplasia of follicles. B—red pulp congestion type: focal or diffuse. C—subjective semi-quantitative score (0 = normal, 1 = discrete, 2 = moderate, and 3 = intense) according to the presence of: red pulp congestion, haemosiderin pigment, red pulp reticular cells/macrophages, red pulp polymorphonuclear cells, red pulp plasma cells, red pulp lymphocytes. D—a quantitative analysis was done using a grid-scale with 10 × 10 subdivisions in an area of 0.0625 mm^2^ to count the cell phenotypes and immune cells expressing cytokines in their cytoplasm. Ten fields were counted randomly at 400 times magnification in the two compartments of the spleen (red pulp and white pulp). Evaluations were carried out independently by two pathologists.

### Statistical Analysis

The categorical data are expressed as percentages and we calculated the Pearson Chi-Square and Fischer's Exact Test. The quantitative data are expressed as median and 25th or 75th percentiles of cells per mm^2^. Differences between the medians of the three groups were analyzed using Kruskal-Wallis. Differences among two groups were analyzed by unpaired *t*-test and by nonparametric methods (Mann-Whitney test). Values of *p* < 0.05 were considered statistically significant.

## Results

### Patient Data

Table 3 summarizes leptospirosis patient clinical and laboratorial characteristics. The group was composed mainly by men (82%) which took 4.5–7 days to present to the hospital after symptoms began and had a high APACHE II score (21 to 28). The median APACHE II score was 23.5, which corresponds to a survival rate of 30%, reflecting the severity of the leptospirosis cases (21). In seven cases, the microagglutination test was positive, and the main serovars were the *Icterohaemorrhagiae (5 cases)* and *Copenhageni (3 cases)*.

**Table 3:** Clinical and laboratory findings of 11 patients with severe leptospirosis with severe pulmonary hemorrhage syndrome and shock.

**Demographic and epidemiologic data**		
Age, median (IR)^*^	40	(36–56)
Male gender, *n* (%)^**^	9	(81.8%)
Contact with rodents, *n* (%)	5	(45.4%)
Contact with dirty water, *n* (%)	7	(63.6%)
Co-morbidities (arterial hypertension), *n* (%)	3	(27.27%)
**CLINICAL SYMPTOMS ON ADMISSION**		
Duration of symptoms, median (IR)	6 days	(4.5–7.0)
Fever, *n* (%)	11	(100%)
Muscle pain, *n* (%)	11	(100%)
Jaundice, *n* (%)	11	(100%)
Altered mental state, *n* (%)	6	(54.5%)
Pulmonary hemorrhage, *n* (%)	11	(100%)
Shock, *n* (%)	11	(100%)
**LABORATORY VALUES**		
Positive microagglutination, *n* (%)	7	(63.6%)
Distribution of serovars diagnosed by MAT in seven patients		
*Icterohaemorrhagiae*	5	(71%)
*Copenhageni*	3	(43%)
*Hebdomadis*	2	(30%)
*Canicola*	2	(30%)
*Autumnalis*	2	(30%)
*Javanica*	1	(14%)
*Australis*	1	(14%)
*Cynopteri*	1	(14%)
*Djasiman*	1	(14%)
Positive ELISA IgM, *n* (%)	5	(45.4%)
Positive liver *Leptospira* antigen, *n* (%)	7	(63.6%)
Positive spleen *Leptospira* antigen, *n* (%)	8	(72.7%)
Haematocrit %, median (IR)	28.79	(25.5–30.85)
Leukocytes, cells/mm^3^, median (IR)	1.69 × 10^4^	(1.29–2.4 × 10^4^)
Platelets, cells/mm^3^, median (IR)	5.0 × 10^4^	(4.0–6.3 × 10^4^)
Serum creatinine, mg/dL, median (IR)	6	(4.5–8)
Serum bilirubin, mg/dL, median (IR)	14	(7–27)
AST, mg/dL, median (IR)	134	(44.5–294)
ALT, mg/dL, median (IR)	78	(25–114)
**CLINICAL COURSE**		
Use of vasoactive drugs, *n* (%)	11	(100.0%)
Mechanical ventilation, *n* (%)	11	(100.0%)
Dialysis, *n* (%)	9	(81.8%)
Duration of ICU hospitalization, median (IR)	2 days	(1.5–9.5)
APACHE II score, median (IR)^***^	23.5	(21.0–27.5)

*IR, Interquartile range; ELISA, enzyme-linked immunosorbent assay; AST, aspartate aminotransferase; ALT, alanine aminotransferase; ICU intensive care unit; APACHE II, Acute Physiology And Chronic Health Evaluation*.

* *Leptospirosis group was older than control group (p = 0.029, Mann-Whitney)*.

** *Leptospirosis group had more males than sepsis group (p = 0.007, Pearson Chi-Square)*.

*** *APACHE II score calculated for 8 patients*.

*Patient sera were analyzed by the microagglutination test (MAT) to identify antigens from the main pathogenic serovars of Leptospira sp. Assays were carried out by the Instituto Adolfo Lutz, São Paulo, SP, Brazil, according to a published protocol (23)*.

Septic shock patient data (Table 4) are as follows: 10 individuals with a median age of 57 years (minimum 19, maximum 68; *p* = 0.02 compared to controls, not different from leptospirosis), 20% male (*p* = 0007 compared to leptospirosis; *p* = 0.001 compared to controls). Main comorbidities were: systemic arterial hypertension (80%) and diabetes mellitus (70%). The autopsy showed that sepsis cases had bronchopneumonia (60%), skin and soft tissue infections (40%), pyelonephritis (20%), and intra-abdominal infections (10%). Median intensive care stay was of 7.5 days (1–30 days). All patients were treated with vasoactive drugs and mechanical ventilation. The APACHE II score was high, but calculated in only two cases (25 and 28).

**Table 4:** Clinical and laboratory findings of 10 patients with septic shock.

**Age (y)**	**Sepsis Diagnosis**
16–20	Bilateral pneumonia
21–25	Bilateral pneumonia
46–50	Pulmonary abscess/pneumonia
51–55	*E. faecalis* sepsis
51–55	Urinary infection
56–60	*S. aureus* sepsis
61–65	Purulent cholecystitis
61–65	*P. aeruginosa* urinary infection
61–65	Bilateral pneumonia
66–70	Infected diabetic foot

As for the control group, healthy portions of the spleen were used from trauma patients. There were 12 individuals, with a median age of 27 years (minimum 22, maximum 40), 92% male; 9 patients were admitted due to a motorbike or car collision with a lamppost and 2 cases resulted from falls.

### Spleen Morphology

**Figure 1:**
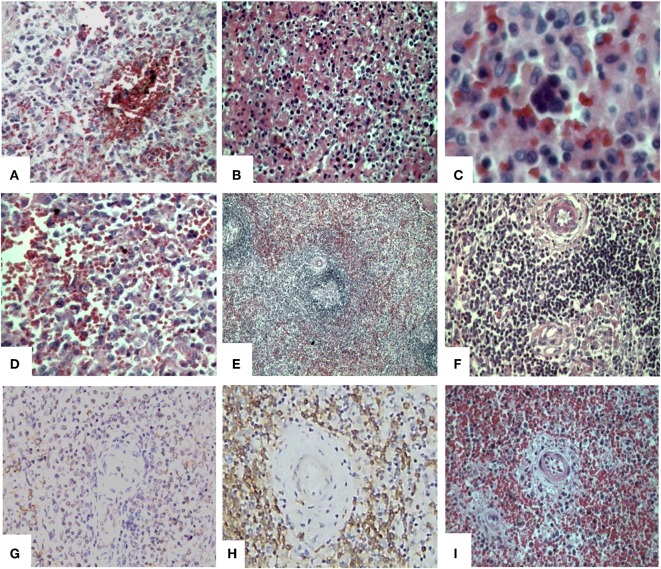
Histological and immunohistochemical findings in the spleen of patients with severe leptospirosis with pulmonary hemorrhage and shock. Spleen from a patient with leptospirosis with **(A)** congestion, hemorrhage and haemosiderin pigments (HE); **(B)** acute splenitis with neutrophils, eosinophils, plasma cells, reticular cells, and macrophages (HE); **(C)** extramedullary haematopoiesis (HE) and **(D)** hypertrophic and hyperplasic reticular cells and macrophages increased in the red pulp (HE). Control patient with **(E)** adequate white pulp, with germinative center (HE); and **(F)** normal T-zone and B-zone (HE); **(G)** Spleen from a leptospirosis patient with positive IH for *Leptospira* antigens in the red pulp. Spleen from a bacterial septic patient with **(H)** white pulp with low TCD8^+^ density by IH stain and **(I)** red pulp congestion and atrophic follicles with depleted T-zone and B-zone(HE). Original magnification 400×, except for **(C)** oil immersion (1000×) and **(E)** 200×. HE, Haematoxylin-Eosin; IH, Immunohistochemistry.

**Figure 2:**
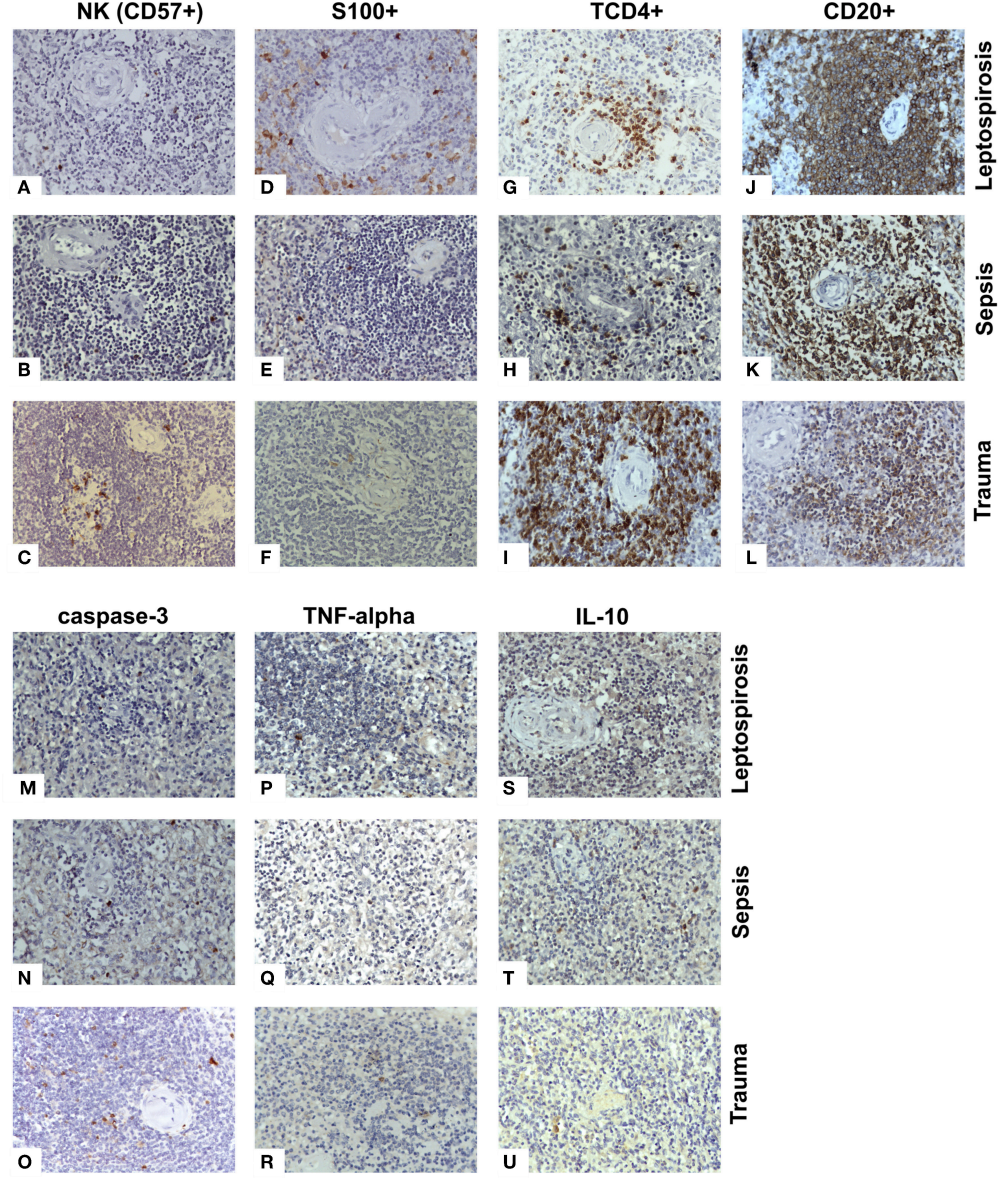
Antigen expression for cell receptors, cytokines and apoptosis in the white pulp of the spleens from fatal cases of Leptospirosis with pulmonary hemorrhage and shock. **(A–C)** NK cells (CD57^+^) were present at lower levels in the leptospirosis and sepsis groups as compared to the control group. **(D–F)** The S100 cells were higher in the leptospirosis group than the sepsis and control groups. **(G–I)** The TCD4^+^ cells were in lower quantity in the leptospirosis and sepsis groups than in the control group. **(J–L)** Both leptospirosis and sepsis groups had more CD20^+^ cells than patients with control in the white pulp. **(M–O)** Cleaved caspase 3 positive (apoptotic) cell levels were lower in the leptospirosis and sepsis groups than in the control group in the white pulp. **(P–R)** TNF-α levels were higher in the leptospirosis group as compared to sepsis and control in both the white and red pulps. **(S–U)** The number of cells expressing IL-10 was greater in leptospirosis and sepsis groups as compared to control in both red and white pulps. Magnification: 400×.

Table 5 and Figures 1, 2 display the results of the histological parameters analyzed. The congestion in the red pulp was more intense and diffuse in the leptospirosis and sepsis groups than in the control group (Figures 1A,I). Pericapsular or sub capsular hemorrhages were more frequent in the control group than in the leptospirosis and sepsis groups. Hemorrhages in the red pulp occurred equally among the groups (Figure 1A).

**Table 5:** Histological and immunohistochemical findings in the spleen of patients with severe leptospirosis (with pulmonary hemorrhage and shock), sepsis or control.

**Pathologic findings**	**Leptospirosis (*n* = 11)**	**Sepsis (*n* = 10)**	**Control (*n* = 12)**	***Overall p*value^***a***^**	***Leptospirosis x sepsis p***	***Leptospirosis x control p***	***Sepsis x control p***
Red pulp congestion^*^							
Absence/discrete	1 (9.1%)	0 (0.0%)	10 (83.3%)	0.0001	0.001	NS	0.0001
Moderate/intense	10 (90.9%)	10 (100%)	2 (16.7%)				
**Congestion type**^@^							
Focal	0 (0.0%)	0 (0.0%)	10 (83.3%)	0.0001	0.001	NT	0.0001
Diffuse	11 (0%)	10 (100%)	1 (8.3%)				
Peri/subcapsular hemorrhage	1 (9.1%)	2 (20.0%)	8 (66.7%)	0.008	0.007	NS	0.038
Red pulp hemorrhage	10 (90.9%)	10 (100%)	8 (66.7%)	0.075	NT	NS	0.068
Red pulp necrosis	1 (9.1%)	3 (30%)	0 (0.0%)	0.093	NT	NS	NS
**Red pulp vessels**							
Necrosis and thrombosis	0 (0.0%)	2 (20.0%)	0 (0.0%)	0.093	NT	NS	NS
Red pulp reticular cells/macrophages^*^							
Normal/discrete	0 (0.0%)	1 (10.0%)	12 (100%)	0.0001	0.0001	NS	0.0001
Moderate/intense	11 (100%)	9 (90.0%)	0 (0.0%)				
Red pulp polymorphonuclear cells^*^							
Normal/discrete	0 (0.0%)	4 (40.0%)	12 (100%)	0.0001	0.0001	0.035	0.003
Moderate/intense	11 (1000%)	6 (60.0%)	0 (0%)				
Red pulp plasma cells^*^							
Normal/discrete	0 (0.0%)	1 (10.0%)	12 (100%)	0.0001	0.0001	NS	00001
Moderate/intense	11 (100%)	9 (90.0%)	0 (0.0%)				
Red pulp lymphocytes^**^							
Normal	0 (0.0%)	0 (0.0%)	11 (91.7%)	0.0001	0.0001	NT	0.0001
Discrete	11 (100%)	10 (100%)	1 (8.3%)				
Red pulp extramedullary haematopoiesis	11 (100%)	4 (40.0%)	0 (0.0%)	0.0001	0.0001	0.004	0.029
**Red pulp haemosiderin pigments**^@^							
Normal	5 (45.5%)	6 (60.0%)	12 (100.0%)	0.064	0.012	NS	NS
Moderate	1 (9.1%)	1 (10.0%)	0 (0.0%)				
Intense	5 (45.5%0)	3 (30.0%)	0 (0.0%)				
**Follicle volume**^@^							
Normal	2 (18.2%)	0 (0.0%)	12 (100%)	0.0001	0.0001	NS	0.0001
Atrophied	9 (81.8%)	10 (100%)	0 (0.0%)				
T-zone depletion	11 (100%)	6 (60.0%)	0 (0.0%)	0.0001	0.0001	0.035	0.003
B-zone depletion	9 (81.8%)	9 (90.0%)	0 (0.0%)	0.0001	0.0001	NS	0.0001
Central artery with signs of activated endothelium	11 (100%)	7 (70.0%)	3 (25.0%)	0.001	0.0001	NS	0.046
**TCD4**^+^ IH^***^							
Red pulp	78.4 (57.6–237.6)	185.6 (90.4–411.2)	624.0 (528.0–846.4)	0.0001	0.0001	0.044	0.0003
White pulp	1835.0 (1367.0–2376.0)	1850.0 (1420.0–2307.0)	5118.0 (3925.0–6040.0)	0.0001	0.0001	NS	0.0001
**TCD8**^+^ IH^***^							
Red pulp	412.8 (260.0–1493.0)	70.40 (49.60–298.40)	583.2 (340.8–761.6)	0.0011	NS	0.0035	0.0008
White pulp	865.6 (509.6–1024.0)	148.0 (62.40–188.0)	835.20 (564.0–1268.0)	0.0001	NS	0.0002	0.0001
**CD20**^+^ IH^***^							
Red pulp	0.73 (0.55–1.08)	0.30 (0.16–0.49)	0.14 (0.08–0.31)	0.0084	0.0082	0.0221	NS
White pulp	8.45 (7.81–9.82)	9.86 (8.13–11.84)	6.01 (5.01–7.46)	0.0001	0.0035	NS	0.0022
NK IH^***^							
Red pulp	19.2 (16.0–26.4)	17.6 (2.4–73.6)	90.4 (73.6–173.6)	0.0003	0.0001	NS	0.0062
White pulp	24.0 (18.4–61.6)	5.6 (0.0–32.8)	135.2 (92.8–198.4)	0.0005	0.0023	NS	0.0022
CD68 IH^***^							
Red pulp	598.4 (153.6–898.4)	1075 (712–1411)	308.0 (224.0–350.4)	0.0007	NS	0.0184	0.0005
White pulp	192.0 (153.6–325.6)	547.2 (404.8–628.0)	148.0 (68.8–184.0)	0.0001	NS	0.0001	0.0001
S100 IH^***^							
Red pulp	686.4 (390.4–1040)	54.4 (12.8–125.6)	4.8 (6.4–8.0)	0.0001	0.0001	0.0003	0.0001
White pulp	214.4 (81.60–288.8)	10.4 (0.8–19.2)	8.8 (1.6–24.0)	0.0001	0.0001	0.0001	NS
Cleaved caspase 3 IH^***^							
Red pulp	4.8 (1.6–17.6)	15.2 (6.4–40.8)	37.6 (8.80–71.20)	0.029	0.016	NS	NS
White pulp	0.0 (0.0–5.6)	3.2 (2.4–14.4)	62.4 (29.6–103.2)	0.0002	0.0006	NS	0.0011
**TNF-α** IH^***^							
Red pulp	22.4 (12.8–32.4)	4.0 (0.8–17.6)	2.4 (0.0–5.6)	0.0021	0.0014	0.0166	NS
White pulp	32.0 (0.0–100)	0.0 (0.0–0.8)	0.0 (0.0–2.4)	0.0001	0.0011	0.0008	NS
**IFN-γ** IH^***^							
Red pulp	0.0 (0.0–4.0)	0.0 (0.0–1.60)	0.0 (0.0–3.20)	0.68	NS	NS	NS
White pulp	0.0 (0.0–0.0)	0.0 (0.0–0.0)	0.0 (0.0–0.0)	0.63	NS	NT	NT
IL1 IH^***^							
Red pulp	0.0 (0.0–0.0)	0.8 (0.0–2.40)	0.0 (0.0–0.0)	0.054	NS	NS	NS
White pulp	0.0 (0.0–0.0)	0.0 (0.0–0.0)	0.0 (0.0–0.0)	0.97	NS	NS	NS
IL2 IH^***^							
Red pulp	0.0 (0.0–24.0)	0.0 (0.0–0.0)	0.0 (0.0–0.8)	0.515	NS	NS	NS
White pulp	0.0 (0.0–3.2)	0.0 (0.0–0.0)	0.0 (0.0–1.6)	0.15	NS	NT	NT
IL6 IH^***^							
Red pulp	3.2 (1.6–11.2)	0.0 (0.0–0.0)	1.6 (0.0–6.4)	0.0021	NS	NT	NT
White pulp	0.0 (0.0–4.0)	0.0 (0.0–0.0)	0.0 (0.0–1.6)	0.11	NS	NT	NT
IL12 IH^***^							
Red pulp	8.0 (4.8–14.4)	0.0 (0.0–0.0)	0.0 (0.0–0.0)	0.0001	0.0031	NT	NT
White pulp	0.0 (0.0–2.4)	0.0 (0.0–0.0)	0.0 (0.0–1.60)	0.076	NS	NT	NS
IL4 IH^***^							
Red pulp	11.2 (0.0–49.6)	0.0 (0.00–0.8)	1.6 (0.0–12.0)	0.034	NS	0.035	0.044
White pulp	0.0 (0.0–24.8)	0.0 (0.0–0.0)	1.6 (0.0–5.6)	0.014	NS	NT	NT
IL10 IH^***^							
Red pulp	33.6 (28.8–64.2)	83.2 (38.4–152.0)	0.0 (0.0–0.8)	0.0001	0.0006	0.024	0.0002
White pulp	20.8 (15.2–52.8)	32.0 (13.6–66.4)	0.0 (0.0–0.8)	0.0002	0.0006	NS	0.0009
**TGFβ** IH^***^							
Red pulp	83.2 (58.4–156.0)	212 (118.4–266.4)	32 (8.0–80.8)	0.003	NS	0.0184	0.0041
White pulp	86.4 (64.0–207.2)	117.6 (71.6–199.2)	81.6 (25.6–124.0)	0.43	NS	NS	NS

@ *Subgroups were added for statistical analysis*.

* *These parameters were regrouped in two categories (normal/discrete or moderate/intense) to do the statistical analysis*.

** *The semi quantitative analysis of the lymphocyte number in the red pulp was regrouped in two categories (normal or discrete) to do the statistical analysis as none of the patients had moderate or intense cord lymphocytic infiltration*.

*** *The data are expressed as median and (in parentheses) the 25th and 75th percentiles of the number of positive cells per square millimeter*.

a *p compares the three groups as determined by Pearson Chi-Square*.

*NS, not significant; NT, not tested*.

In the leptospirosis and sepsis groups, the reticular cells showed pronounced hyperplasia and hypertrophy and there was an increased number of macrophages in the red pulp (Figure 1D).

In the cords, neutrophil, eosinophil, plasma cell and lymphocyte numbers were increased in leptospirosis and sepsis groups in comparison with the control group.

In response to stress, including bacterial infections, stromal haematopoietic stem cells around the sinusoids of the spleen can be activated, enabling extramedullary haematopoiesis to occur (7, 24). Although this observation is not directly associated with the immune response, we would like to mention that we describe, for the first time, that extramedullary haematopoiesis occurs in leptospirosis. It occurred more frequently in leptospirosis patients than in the sepsis group and was not observed in control patients, as expected (Figure 1C). We believe this observation may be associated with the *Leptospira* virulent factors or with the fact that leptospirosis patients were younger and may have had more responsive precursor cells.

The follicles of the leptospirosis and sepsis (Figure 1I) groups were atrophic, whereas those of the control group were normal (Figure 1E).

The T-cell and B-cell-dependent regions had a lower cellular density in the leptospirosis and sepsis groups as compared to the control group (Figures 1F,I).

The endothelium of the central artery showed signs of activation in the leptospirosis and sepsis groups in comparison with the control group (Figures 1F,I). Artery activation is characterized by: tumefaction of the endothelial cell (cubical aspect as compared to normal flat cells) and arterial wall oedema.

### Immunohistochemistry for Cytokines and Cell Markers

The *Leptospira* antigen was positive in the spleen of 8 out 11 of the leptospirosis patients (72.7%) (Figure 1G).

NK cells (CD57^+^) were present at lower levels (≤20% of control) in the leptospirosis and sepsis groups as compared to the control group (Figures 2A–C).

Dendritic S100^+^ cells levels were increased more than 10-fold in the red pulp of both leptospirosis and sepsis groups as compared to control and the levels found in leptospirosis tissues were higher than those in the septic tissues. However, in the white pulp, S100 cells were higher only in the leptospirosis group (Figures 2D–F). The changes in dendritic cell numbers were the most extreme findings regarding cell data in our study.

Cleaved caspase 3 positive (apoptotic) cell levels were lower in the leptospirosis and sepsis groups than in the control group in the white pulp (Figures 2M–O). Indeed, no active caspase 3-positive cells were found in the leptospirosis group (compared to 62.4 cells/mm^2^ in the control group). Apoptosis was also significantly lower in the leptospirosis group compared to the sepsis group in the red pulp.

CD68^+^ cells were found at higher levels in the sepsis group as compared to leptospirosis and control groups.

The TCD4^+^ cells were in lower quantity in the leptospirosis and sepsis groups than in the control group (Figures 2G–I). More TCD4^+^ cells were found in the red pulp of the sepsis group than in that of the leptospirosis group.

TCD8^+^ cell numbers in the red and white pulps were lower in the sepsis group than in the leptospirosis (Figure 1H) and control groups.

Both leptospirosis and sepsis groups had more CD20^+^ cells than patients with control in the white pulp (Figures 2J–L). The leptospirosis group had more CD20^+^ cells than the sepsis and control groups in the red pulp.

There was a minimum expression of Th1 cytokines (IFN-γ, IL-1, IL-2, and IL-6) in all experimental groups, with no statistical difference. An exception was IL-12, with a significantly higher expression in the red pulp of the leptospirosis patients as compared to the control group. We have added immunohistochemistry positive controls presented in Supplementary Figure 1 to indicate that low expression was not a result of incomplete staining.

TNF-α levels were higher in the leptospirosis group as compared to sepsis and control groups in both the white and red pulps (Figures 2P–R). In fact, no TNF-α-positive cells were found in sepsis and control patients in the white pulp, whereas 32 cells/m^2^ were found in leptospirosis samples.

Control and leptospirosis groups had similar distribution of IL-4 in both white and red pulps and this expression was higher than that of the septic group in the red pulp.

The number of cells expressing IL-10 was greater (more than 10-fold, *p* < 0.0005, one of the greatest changes found in our study regarding cytokines) in leptospirosis and sepsis groups as compared to control in both red and white pulps (Figures 2S–U).

As for TGF-β, its expression was similar in all three groups in the white pulp, whereas levels were higher in the sepsis group compared to the other two groups in the red pulp.

## Discussion

We compared severe leptospirosis with sepsis—infection with Gram positive or Gram-negative bacteria—and control samples. This is the first report to explore the immune aspects of severe leptospirosis with shock and SPHS.

We focused our study on the spleen, where there is heavy splenocyte death among the lethal cases of septic shock (9) and because of changes in immune system response reflected in this organ.

### Patient Data

Leptospirosis patients have a very characteristic demographic: they are mostly young and male. We found it difficult to find corresponding sepsis patients, that were older and only 20% male, and controls, that were younger than the leptospirosis group. The most common serovars identified in the present cases were *Icterohaemorrhagiae* and *Copenhageni*, of the *Leptospira interrogans* serogroup, which are the most prevalent in the State of São Paulo and are associated with severe cases of leptospirosis (23).

Some authors believe that antibiotic treatment in severe cases of leptospirosis is unnecessary because, at this stage, it is likely that all bacteria have already been eliminated and that focus should be on treating organ damage due overreacting immune and inflammatory response (25). We describe that the *Leptospira* antigen is still present in the spleen at time of death, which implies that early antibiotic treatment probably could have saved some patients and should be prescribed in cases of severe leptospirosis. However, we should point out that the time until admission was found to have a median of 5 days. It is likely that this delay in treating patients with antibiotics (ceftriaxone) and intravenous hydration was an important factor in mortality. Our results indicate that health providers should be trained to recognize leptospirosis as early as possible and start antibiotic treatment to avoid sepsis.

### Spleen Morphology

Our results demonstrate a strong similarity in histological aspects between leptospirosis and septic spleens of patients, as opposed to controls, displaying a pattern of acute splenitis as found by other authors (26–28). The endothelial cells of the central artery had histological signs of activation in leptospirosis without necrosis. In contrast, the sepsis group had endothelial activation plus, in two cases, endothelial necrosis and thrombosis. Increased plasma levels of sE-selectin and Von Willebrand factor, possibly derived from endothelial cells, have been previously described in leptospirosis patients and higher sE-selectin levels were associated with lower mortality (29).

### Leptospira Antigen in Spleen

The *Leptospira* antigen showed strong labeling in a granular pattern widely distributed in the red and white pulps, both intracellularly (macrophages, reticular cells, endothelial cells) and extracellularly, indicating that these bacteria probably have an important role in the pathogeny of severe leptospirosis. This is the first study to show that the *Leptospira* antigen is present in the spleen of patients with leptospirosis, although this antigen has been previously found in the liver, kidneys, heart, lungs, and skeletal muscle (3, 30–32).

As discussed earlier, the presence of *Leptospira* and signs of immunosuppression in later and severe stages of infection corroborate that antibiotic treatment should occur at all stages of the disease as prescribed for other types of sepsis (33) and proven to be beneficial in animal models (34).

### Innate Immune Response

Although our study was carried out evaluating immune response in both red and white pulps, we consider that the red pulp is a transition zone and will focus our discussion on results obtained in the white pulp.

We report, for the first time, that patients with severe leptospirosis have a compromised innate immune response associated with a decrease in NK cells and low levels on IFN-γ. The decrease in NK levels was also found in sepsis. Low NK cell count was previously found in an *ex vivo* report for leptospirosis (35), although *Leptospira* are known to stimulate these cells *in vitro*; NK levels at early stages of the disease probably correlate with its outcome (36, 37), as found in sepsis (38).

We found a >10-fold increased number of dendritic cells in leptospirosis and in sepsis patients. In leptospirosis, an *ex vivo* study found that dendritic cells proliferate and secrete a high quantity of Th1 cytokines and a low quantity of IL-10 (39). Reports for sepsis (10, 40) show a loss of dendritic cells in the spleen due to apoptosis as opposed to our results. This can be explained by our different methodology: we employed a broad marker to detect dendritic cells—S100, while other authors used specific tissue markers for follicular cells (CD21 and FDC-M1) (10, 40). The S100 protein allows us to detect myeloid cells, including interdigitating dendritic cells, Langerhans cells and follicular dendritic cells (41). We chose this marker because of its availability and because it allows us to detect dendritic cells in both red and white pulp. We also believe a high number of dendritic cells is possible in the leptospirosis group and can be attributed to the virulence factors, young age and predominant male gender.

Regarding inflammation, we believe patients had suffered from an intense inflammatory response early during infection since both leptospirosis and sepsis patients presented with severe tissue lesions including nephritis, interstitial pneumonitis, pulmonary hemorrhage, hepatopathy, and muscular lesions. As mentioned above, histological analysis also indicated splenitis probably caused by an intensive inflammatory response to the infective agents.

### T and B Lymphocytes—Acquired Immune Response

Both leptospirosis and sepsis patients showed clear follicle atrophy in the white pulp, with low cellular density in the T- and B-cell dependent regions. This is a strong indication of the inhibition of the adaptive immune response. TCD4^+^ levels in the leptospirosis and sepsis groups were significantly lower as compared to controls. The depletion of TCD4^+^ cells in leptospirosis appears to be essential for the host's homeostasis (42, 43).

We have no data to indicate how TCD4^+^ cells are lost in leptospirosis, though it is likely by apoptosis, as found in animal models (44) and in spleens of patients with septic shock (8–10). However, we found a *decreased* level of apoptosis in the sepsis and leptospirosis patients as compared to controls. It is possible that our results are due to the methodological approach: we used antibodies against activated caspase-3, whereas other authors used anti-caspase 9 or the TUNEL assay, which may label necrotic cells (45). Another possibility is that there is a massive apoptotic event during earlier stages of infection, which is completed by the later stages—and cannot be detected—also suggesting that no new lymphocytes migrate to the spleen. Other forms of cell death or control of cell population by inhibition of proliferation are other possible explanations for the low amount of immune cells found in sepsis and leptospirosis, especially since we found low levels of proliferation stimulants (i.e., NK cells and cytokines) (46).

Regarding CD20^+^ B lymphocytes, they were found in increased numbers in both infectious groups as compared to control which may be associated with a humoral response to the infectious agents.

### Cytokine Expression in the Spleen

We showed that TNF-α levels are increased in leptospirosis in comparison with the sepsis and control groups, in spite of the lack of regulation of other Th1 cytokines. TNF-α is the most studied cytokine in leptospirosis and has been found to be a marker for clinical outcome (47, 48).

We found that IL-1, IL-2, IL-4, IL-6, IL-12, and IFN-γ levels were very low or undetectable, with no major changes among patient groups, confirming previous studies (48–53). IL-12 levels were expected to be high in leptospirosis, based on *in vitro* and animal studies (39, 51, 54). Our results showed elevated expression of IL-12 in leptospirosis in the red pulp, but since it was absent from the white pulp, the overall result is probably of immunosuppression. The low secretion of IFN-γ during the immunoparalysis phase of sepsis is a key aspect of this syndrome and is associated with its outcome (55).

We found increased levels of IL-10 in both sepsis and leptospirosis groups indicating an immunosuppressed environment and confirming previous studies in other tissues (51, 56, 57).

TGF-β levels were also increased in the spleens of leptospirosis and sepsis patients, as described in kidneys of hamsters infected with *Leptospir*a (57).

### Overall Th1 vs. Th2 Response: Immunoparalysis in Severe Infections

We conclude from our data and its corroboration by other studies that there is immunoparalysis in leptospirosis as also occurs in sepsis. However, as our reviewers have pointed out, the data is restricted to a late time point (after death), a single tissue (spleen) and morphological analysis of preserved tissues—and therefore that it is hard to be certain of the immunological processes that occurred in the patients. Therefore, the following analysis should be viewed with caution.

The *in situ* decrease (or lack of expression) in Th1 pro-inflammatory cytokines (IL-6, IFN-y, IL-1β, IL-2r, and IL-12) and the increase in IL-10 indicate that there is an inhibition of the innate and acquired immunity during the acute and severe phases of leptospirosis and sepsis. Previous data obtained *in vitro* or in patient serum indicate that there is an initial Th1 response in leptospirosis (36, 37, 39, 47, 58–62) as opposed to our findings in later stages of the disease. This may indicate that in later stages of severe leptospirosis there is an immune system burnout or some sort of inhibition of immune response triggered by bacteria.

Human patients and animal models show that sepsis is associated with an immunosuppressive state (55, 63–65) corroborating that severe leptospirosis is similar to sepsis.

Overall, our study shows that there is an “immunoparalysis” in severe leptospirosis and sepsis: low quantity of NK and TCD4^+^ cells, low expression of IFN-γ, IL-12, IL-6, IL-1 and IL-2 and high expression of IL-10. The successful immune response to leptospirosis appears to depend on the secretion of Th1 pro-inflammatory cytokines (36, 37, 59, 61, 66). We believe that the host's response to the infectious agent needs to hit a sweet spot: not too high (such as with cytokine storms) and not too low, as in immunoparalysis (63, 64, 67). Immunoparalysis has been shown in sepsis, where it favors the development of new infections and morbidity (63, 68). Sepsis associated with septic shock has been correlated with a Th2 response (55, 64, 69). Indeed, in hamster models of *Leptospira* infection, it has been shown that there is an initial Th1 response followed by a predominance of high IL-10 at later stages of the infection (51, 56, 57).

We also found that there appears to be a lack of interaction between innate and adaptive immunity when comparing the increase in dendritic cells, but decrease in IL-2 and IL-12 expression—in dendritic cells. Had the levels of these cytokines increased, then they should have been able to stimulate other Th1 cells (70).

We hypothesized that severe leptospirosis with shock has some elements of immunosuppression-like septic shock as we have demonstrated in the spleen. Leptospirosis patients should be included in clinical trials for sepsis treatment and biomarker discovery as well as sepsis caused by Gram +/– bacteria (9, 71, 72).

Our results indicate that an immunomodulatory treatment may aid in patient recovery, controlling the initial immune response in order to prevent immune system burnout or stimulating the immune system at later stages (8, 12, 73). Another practical aspect of our results is that it suggests that immunoparalysis markers can be useful in patient diagnosis and to aid treatment (63).

Our study has some limitations: it was based on retrospective data recovered from patient files. This has drawbacks because some clinical data was not collected at the time of admission or during treatment (especially for several sepsis cases), which restricts our analysis. Also, because Brazilian law requires that autopsies be carried out at least 6 h after death—the median time until autopsy was 8 h in our study—some sample autolysis may have happened, with loss of antigens, although HE staining shows no signs of tissue degradation and staining controls were positive. Since we based our study on patients that died from leptospirosis or sepsis, we do not have spleen data for mild cases, where there was recovery. We also do not have spleen samples from severe cases before death (at earlier stages of the severe disease). Overall, we based our study on morphological aspects of the spleen and do not have data relating to systemic immune response, such as circulating immune cell types and circulating cytokines and other inflammatory markers. We also designed our study based on broad cell markers and not on more specific markers (for example, CD21 for dendritic follicular cells).

## Conclusion

To the best of our knowledge, this is the first study evaluating immune response in severe leptospirosis-like septic shock. Our research demonstrates that severe leptospirosis behaves like septic shock caused by Gram +/– bacteria regarding: clinical/biochemical presentation and spleen histopathological characteristics. Given that we found an intense and diffuse *Leptospira* antigen distribution and an immune response characterized by a low Th1/Th2 ratio in the spleen, patient treatment should include antibiotics and immunomodulators to reduce mortality.

## Ethics Statement

This study is a retrospective histological evaluation of the spleen samples from three different groups of patients (leptospirosis, sepsis and trauma) obtained by necropsy (leptospirosis and sepsis) or by surgery (trauma) from the Department of Pathology of the Medical School of the University of São Paulo. The records of the Death Verification Service of the Department of Pathology were reviewed to identify all deaths due to severe leptospirosis with LPHS and shock during the period between January 1st 1988 and January 1st 2005. The project was approved by the Ethical Committee and Research board of the Clinical Hospital of the University of São Paulo Medical School (registered by CAPPesq ICHC n° 0537/06).

## Author Contributions

AD-N performed the autopsies, conceived and designed the analysis, collected the data, performed the analysis, and wrote the paper. JC collected the data and revised the paper. CP collected the data, performed the analysis, and wrote the paper. FS treated the patients during the hospitalization, performed the analysis, and wrote the paper. AN treated the patients during the hospitalization, collected the data, performed the analysis, and revised the paper. MD performed the autopsies, conceived and designed the analysis, collected the data, performed the analysis, and wrote the paper.

### Conflict of Interest Statement

The authors declare that the research was conducted in the absence of any commercial or financial relationships that could be construed as a potential conflict of interest.

## Supplementary Material

Supplementary Figure 1 Positive immunohistochemical controls for low-expressing cytokines. Expression of the following cytokines was found to be very low in all groups (leptospirosis, sepsis and control). This figure shows positive staining in control spleen: **(a)** IL-1β; **(b)** IFN-γ; **(c)** IL-2; **(d)** IL-6. Magnification: 400× **(a,c)**; 200× **(b,d)**.
