# Supplementary figures and images for: Severe Leptospirosis Features in the Spleen Indicate Cellular Immunosuppression Similar to That Found in Septic Shock
Source: Front Immunol. 2019 Apr 30;10:920. doi: 10.3389/fimmu.2019.00920 (PMC6503108; doi:10.3389/fimmu.2019.00920)

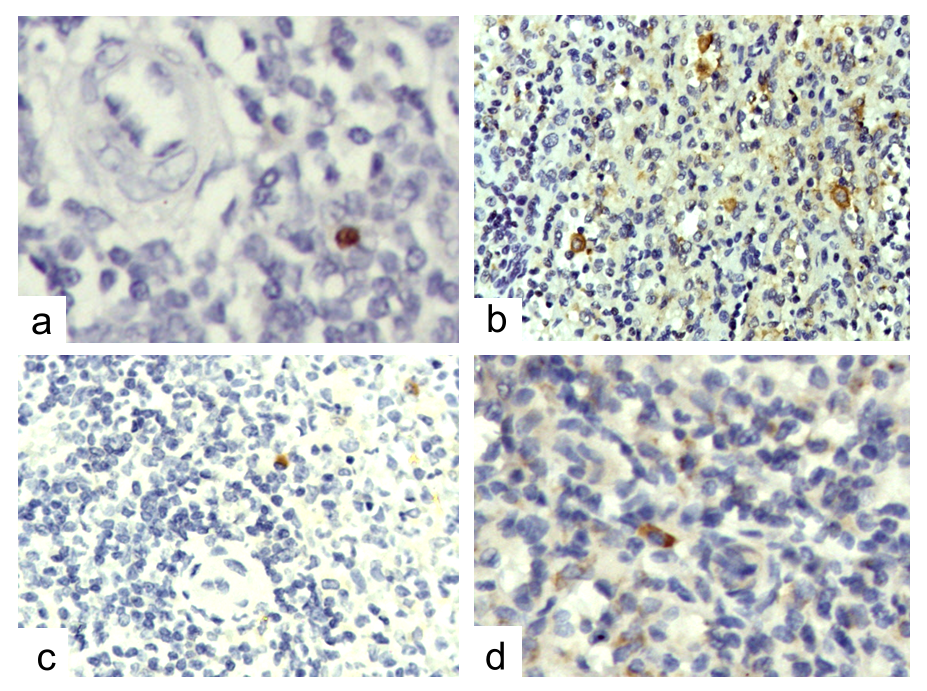

Supplement: Supplementary Figure 1 — Positive immunohistochemical controls for low-expressing cytokines. Expression of the following cytokines was found to be very low in all groups (leptospirosis, sepsis and control). This figure shows positive staining in control spleen: (a) IL-1β; (b) IFN-γ; (c) IL-2; (d) IL-6. Magnification: 400× (a,c); 200× (b,d). [file Image_1.TIF]
